# Comment on “Transmission Model of Hepatitis B Virus with the Migration Effect”

**DOI:** 10.1155/2015/469240

**Published:** 2015-08-30

**Authors:** Abid Ali Lashari

**Affiliations:** ^1^School of Natural Sciences, National University of Sciences and Technology, H-12, Islamabad 44000, Pakistan; ^2^Department of Mathematics, Stockholm University, 10691 Stockholm, Sweden

## Abstract

Some consequences of erroneous results concerning eigenvalues in the recent literature of mathematical biology are highlighted. Furthermore, an improved stability criterion and the true value of the basic reproduction number is presented.

## 1. Introduction

Stability analysis of a mathematical model describing the dynamics of a problem in biology requires a knowledge of the eigenvalues of the Jacobian matrix associated with the matrix [1]. The Routh-Hurwitz criteria give necessary and sufficient conditions for the eigenvalues to lie in the left half of the complex plane. However, recent literature in mathematical biology contains instances of authors establishing stability of the Jacobian matrix by using erroneous results concerning eigenvalues of a matrix. One of the results is stated below.

1. Eigenvalues of a matrix are invariant under elementary row [or column] operations. See, for example, Khan et al.'s Theorems 1 and 2.

## 2. Falseness of the Above Statement

The example occurs in the proof of Theorems 1 and 2 of Khan et al. [2]. The theorem states the following:

(1) * For R*_0_ ≤ 1*, the disease-free equilibrium of the system (3) about an equilibrium point D*_0_ = (*S* ^0^, 0,0, 0,0)* is locally asymptotically stable if Q*_1_(*δ* + *δ*_0_ + *p*)(*δ* + *γ*_1_) > *βγ*_1_(*δπ* + *δ*_0_)*; otherwise, the disease-free equilibrium of system (3) is unstable for R*_0_ > 1.
(2) * For R*_0_ > 1*, the endemic equilibrium D* ^*∗*^ *of system (3) is locally asymptotically stable, if the following conditions hold:*
  (1) $$\begin{array}{c} \gamma_{1}Z_{2}\delta_{0}S^{*2}>\gamma_{1}\beta^{2}\kappa ,\\ G_{1}>G_{2}; \end{array}$$
 
*otherwise, the system is unstable.*

In order to prove these results, they performed elementary row operation for the Jacobian matrix *J*_0_(*ζ*) (9) at the disease-free equilibrium *D*_0_ and obtained matrix *J*_0_(*ζ*) (10) (similarly, by elementary row operation for the Jacobian matrix *J* ^*∗*^(*ζ*) (14) at *D* ^*∗*^ and obtaining the matrix *J* ^*∗*^(*ζ*) (15)). Then, they analyse matrices (10) and (15) obtained after elementary row transformation from (9) and (14), respectively, to show that all the eigenvalues of (9) and (14) are negative from which they concluded the above assertions of their theorem. This reasoning would have been valid if elementary row transformation preserved eigenvalues, which, however, does not as shown below.

The eigenvalues for matrices (9) and (10) (similarly of matrices (14) and (15)) in [2] are not the same as they violate the well known criteria that the sum of the eigenvalues of a matrix is the same as the trace of that matrix. Now, the difference of the traces of matrices (9) and (10) is

(2) $$\begin{array}{c} -\left(\delta +\gamma_{1}\right)-Q_{1}-\left(\delta +\gamma_{3}\right)\neq 0. \end{array}$$

By the same reasoning, it can be easily seen that the difference of the traces of matrices (14) and (15) is also not zero. The eigenvalues would have been the same if the difference between the trace of the original and the trace of the matrix obtained after row transformation equals zero, which, however, does not. Clearly, the eigenvalues may change after an elementary row transformation. The above statement may hold in special cases but is false in general.

Moreover, the true value of the basic reproduction number *R*_0_ of system (3), which measures the average number of new infections generated by a single infected individual in a completely susceptible population, is given by

(3) $$\begin{array}{c} R_{0}=\frac{\gamma_{1}\beta S^{0}\left(\delta +\gamma_{3}\right)+q\gamma_{1}\gamma_{2}\left(\beta \kappa S^{0}+\delta \pi \eta \right)}{\left(\delta +\gamma_{3}\right)\left(\delta +\gamma_{1}\right)Q_{1}}. \end{array}$$

Now, we will show that the local stability of the disease-free equilibrium is completely determined by *R*_0_ and present an improved stability result below.

**Theorem 1 .**

The disease-free equilibrium of model (3) in [2] is locally asymptotically stable if *R*_0_ < 1 and unstable if *R*_0_ > 1.

**Proof**

The characteristic equation of the Jacobian matrix (9) in [2] is given by

(4) $$\begin{array}{c} \left(\lambda +\delta_{0}+\delta +p\right)\left(\lambda +\mu_{1}+\mu_{2}+\delta \right)\left(\lambda^{3}+a_{1}\lambda^{2}+a_{2}\lambda +a_{3}\right)=0, \end{array}$$

where

(5) $$\begin{array}{c} a_{1}=Q_{1}+2\delta +\gamma_{1}+\gamma_{3},\\ a_{2}=\left(\delta +\gamma_{1}\right)Q_{1}+\left(\delta +\gamma_{3}\right)\left(Q_{1}+\delta +\gamma_{1}\right)-\gamma_{1}\beta S^{0},\\ a_{3}=\left(\delta +\gamma_{3}\right)\left(\delta +\gamma_{1}\right)Q_{1}\left(1-R_{0}\right). \end{array}$$

Two of the roots of the characteristic equation (4), *λ*_1_ = −*δ* − *δ*_0_ − *p* and *λ*_2_ = −*μ*_1_ − *μ*_2_ − *δ*, have negative real parts. The other three roots can be determined from the cubic term in (4). Using (5), direct calculations shows that

(6) $$\begin{array}{c} a_{1}a_{2}-a_{3}=\left(Q_{1}+2\delta +\gamma_{1}+\gamma_{3}\right)\left(\underset{︸}{\left(\delta +\gamma_{1}\right)Q_{1}-\gamma_{1}\beta S^{0}}\right)+\left(Q_{1}+2\delta +\gamma_{1}+\gamma_{3}\right)\left(\delta +\gamma_{3}\right)\left(Q_{1}+\delta +\gamma_{1}\right)-\left(\delta +\gamma_{3}\right)\left(\delta +\gamma_{1}\right)Q_{1}+\gamma_{1}\beta S^{0}\left(\delta +\gamma_{3}\right)+q\gamma_{1}\gamma_{2}\left(\beta \kappa S^{0}+\delta \pi \eta \right)=\left(Q_{1}+2\delta +\gamma_{1}+\gamma_{3}\right)\left(\underset{︸}{\left(\delta +\gamma_{1}\right)Q_{1}-\gamma_{1}\beta S^{0}}\right)+\left(\delta +\gamma_{3}\right)\left[\left(2\delta +\gamma_{1}+\gamma_{3}\right)\left(Q_{1}+\delta +\gamma_{1}\right)+Q_{1}^{2}+\gamma_{1}\beta S^{0}\left(\delta +\gamma_{3}\right)+q\gamma_{1}\gamma_{2}\left(\beta \kappa S^{0}+\delta \pi \eta \right)\right]. \end{array}$$

The term under brace is greater than zero if *R*_0_ < 1. Hence, *a*_1_ *a*_2_ − *a*_3_ > 0. Thus, by Routh-Hurwitz criteria, the DFE of system (3) in [2] is locally asymptotically stable about the point *D*_0_ = (*S* ^0^, 0,0, 0,0) if *R*_0_ < 1. Therefore, the extra condition *Q*_1_(*δ* + *δ*_0_ + *p*)(*δ* + *γ*_1_) > *βγ*_1_(*δπ* + *δ*_0_) is not required and the local stability of the disease-free equilibrium is completely determined by *R*_0_.

## 3. Conclusion

This paper has pointed out some technical problems in the results in [2] and has presented the corrected version of the corresponding result and the true value of the basic reproduction number. Studies of mathematical models of the spread of hepatitis B virus have great impact on health authorities' planning and allocation of funds to control the spread of infectious diseases. The effective control decisions of the disease have an important role in the combat of the disease and will be very useful for the public as well as the funding agencies. However such resources are likely to go to waste if scientific studies which purport to guide them are based on faulty theoretical basis. The conclusion based on the model proposed by Khan et al. [2] may not be valid and hepatitis B may still be far from reaching its equilibrium from the community. A wrong mathematical result published in a respectable journal, if left unchallenged, is usually accepted by young research workers as gospel. It is likely to corrupt the scientific literature with growing speed in a manner like the spreading of an infectious disease.
